# Plasma Extracellular Vesicles Enhance HIV-1 Infection of Activated CD4^+^ T Cells and Promote the Activation of Latently Infected J-Lat10.6 Cells *via* miR-139-5p Transfer

**DOI:** 10.3389/fimmu.2021.697604

**Published:** 2021-06-24

**Authors:** Isobel Okoye, Lai Xu, Olaide Oyegbami, Shima Shahbaz, Desmond Pink, Priscilla Gao, Xuejun Sun, Shokrollah Elahi

**Affiliations:** ^1^ Division of Foundational Sciences, School of Dentistry, Faculty of Medicine and Dentistry, University of Alberta, Edmonton, AB, Canada; ^2^ Department of Oncology, Faculty of Medicine and Dentistry, Edmonton, AB, Canada; ^3^ Department of Medical Microbiology and Immunology, Faculty of Medicine and Dentistry, University of Alberta, Edmonton, AB, Canada; ^4^ Li Ka Shing Institute of Virology, University of Alberta, Edmonton, AB, Canada

**Keywords:** HIV, extracellular vehicles (EVs), HIV latency, CD4^+^ T cells, microRNAs, T cell activation, PD-1/PD-L1

## Abstract

HIV latency is a challenge to the success of antiretroviral therapy (ART). Hence patients may benefit from interventions that efficiently reactivate the latent virus to be eliminated by ARTs. Here we show that plasma extracellular vesicles (pEVs) can enhance HIV infection of activated CD4^+^ T cells and reactivate the virus in latently infected J-Lat 10.6 cells. Evaluation of the extravesicular miRNA cargo by a PCR array revealed that pEVs from HIV patients express miR-139-5p. Furthermore, we found that increased levels of miR-139-5p in J-Lat 10.6 cells incubated with pEVs corresponded with reduced expression of the transcription factor, FOXO1. pEV treatment also corresponded with increased miR-139-5p expression in stimulated PD1+ Jurkat cells, but with concomitant upregulation of FOXO1, Fos, Jun, PD-1 and PD-L1. However, J-Lat 10.6 cells incubated with miR-139-5p inhibitor-transfected pEVs from HIV ART-naïve and on-ART patients expressed reduced levels of miR-139-5p than cells treated with pEVs from healthy controls (HC). Collectively, our results indicate that pEV miR-139-5p belongs to a network of miRNAs that can promote cell activation, including latent HIV-infected cells by regulating the expression of FOXO1 and the PD1/PD-L1 promoters, Fos and Jun.

## Introduction

The battle to overcome the scourge of HIV infection and AIDS worldwide is far from over despite advances in antiretroviral therapy (ART). Persistent challenges are the maintenance of virus control, high CD4^+^ T cell count and the optimisation of ART regimens (1). Moreover, intensive research to eliminate HIV reservoirs- latently-infected resting CD4^+^ T cells, are ongoing (2, 3). The occurrence of immune activation and ensuing low-grade inflammation results in increased risk of non-AIDS-defining comorbidities, which impact the quality of life of patients (4). Hence, strategies that will target latently-infected cells, and thus supplement current antiretroviral approaches are warranted.

Extracellular vesicles (EVs) are secreted by all cells and harbour various proteins, lipids and nucleic acids, reminiscent of the cell of origin and designated function (5). Consequently, as intercellular communicators, EVs (30-1000 nm in size), can influence the function and properties of recipient cells, including cells of the immune system (6). In ‘healthy individuals’ EVs found in the circulation are likely to originate from activated and apoptotic cells and participate in physiological and pathological processes (7). Studies have shown that EVs are copiously released into the circulation by HIV-infected cells such as CD4^+^ T cells, monocytes and dendritic cells in response to activation, stress or apoptosis (8). Furthermore, a study has shown that the quantity of plasma EVs correspond with increased viral pathogenesis and fail to decline in response to ART (9). The sustained abundance of plasma EVs in HIV patients was found to correlate with high levels of extravesicular *Nef* protein, concomitant immune activation and low CD4^+^ T cell count (9). Thus, similar to tumour exosomes, plasma EVs may be integral to HIV pathogenesis, spread and the modulation of viral latency. EVs have immunoregulatory properties, which stem mainly from the originator secreting cell and are attributed to cargoes such as MHC I, MHC II, heat-shock proteins, stimulatory receptors and chemokine receptors (6). Hence, EVs have the potential to facilitate the reactivation of latent HIV- infected cells.

MicroRNAs (miRNAs), a class of small, non-coding RNA molecules between 20-22 nucleotides in length, are harboured in EVs in appreciable quantities and play a role in regulating the biological processes of recipient cells by binding to their target genes (10). A study by Hubert et al. showed that in HIV ART-naïve patients, EV abundance inversely-correlates with miR-155 and miR-223 expression, CD4/CD8 T cell ratio and CD4^+^ T cell count (11). Such observations indicate that the EV miRNA composition reflects HIV load and may contribute to disease pathogenesis, viral spread or control (12).

Here, in our quest to evaluate how plasma EVs (pEVs) from HIV patients modulate T cell function in comparison to pEVs from healthy controls (HCs), we carried out a proof-of-concept study to determine whether pEVs can activate latent HIV, using J-Lat 10.6 cells. We found that pEVs from HIV-positive and HCs induced increased expression of miR-139-5p in latently infected J-Lat 10.6 cells which corresponded with virus reactivation and CD38 expression. Furthermore, the expression of miR-139-5p by pEV-treated J-Lat 10.6 cells inversely correlated with the levels of FOXO1 expressed by these cells. In contrast, the expression of miR-139-5p by stimulated PD1+ Jurkat cells pre-incubated with pEVs was associated with increased expression of FOXO1, Fos, Jun, PD-1 and PD-L1. These observations indicate that a network of miRNAs, including miR-139-5p, regulate the expression of FOXO1 and the stringency of this relationship may be dependent on the implications of FOXO1 activity. Interestingly, targeting of miR-139-5p harboured by HIV pEVs resulted in reduced expression of miR-139-5p and increased expression of Fos and Jun, but not FOXO1 by pre-treated J-Lat 10.6 cells. These observations indicate that pEVs, particularly from HIV patients, can induce increased expression of miR-139-5p, which can promote the activation of J-Lat 10.6 cells by targeting FOXO1 and the transcription of PD-1 and PD-L1.

## Materials and Methods

### Study Population and Ethics Statement

Blood samples from 39 HIV-infected individuals (16 HIV ART-naïve and 23 patients on ART) (**Supplementary Table 1**) and 45 HIV, HCV, and HBV seronegative HCs were used for this study. The appropriate Institutional Review Boards at the University of Alberta approved these studies with protocol numbers Pro000046064 and Pro000070528. All study participants gave written informed consent to participate in this study.

### Blood Samples and Plasma Isolation

HIV patients were grouped according to the following criteria: naïve (HIV-infected, antiretroviral therapy (ART)-untreated) and ART (HIV-infected, ART-treated) (13). Peripheral blood samples were collected in EDTA tubes and centrifuged at 1200 rpm for 10 mins to separate the plasma fraction. Plasma samples were aliquoted and stored at -80°C until use. Peripheral blood mononuclear cells (PBMCs) obtained by leukapheresis of healthy donors and frozen were thawed for EV-uptake assays.

### Isolation of EVs and EV RNA

Plasma samples were thawed and centrifuged at 16,000 x g for 15mins at 4°C to pellet cells, debris and platelets. Extracellular vesicles (EVs) were isolated using the miRCURY^®^ Exosome Serum/Plasma Kit (Qiagen) or the exoEasy Maxi Kit (Qiagen) according to manufacturer’s instructions. Protein concentrations of isolated EV samples (in µg/ml) were determined using the Pierce BCA Protein Assay Kit (Thermo Fisher) according to manufacturer’s instructions. The number of EVs per 100 µg of protein was quantified using the EXOCET Exosome Quantitation Kit (System Biosciences).

pEV RNA was isolated using the exoRNeasy Serum Plasma Kit (Qiagen), according to manufacturer’s instructions. The miRNeasy Serum/Plasma Spike-In Control (1.6 x 10^8^ copies/µl) was added to samples during RNA isolation. RNA was isolated from equal volumes of plasma samples from healthy controls and HIV patients.

### pEV Characterisation

Particle size and concentration of pEVs were determined *via* nanoparticle tracking analysis (NTA) using Nanosight LM10 equipped with 405nm laser, 60mW, software version 3.00064. Briefly, samples were diluted 1000- fold using phosphate buffered saline that had been filtered through 0.1micron filter and analyzed in triplicate for 60 seconds per replicate with a count range of 20-100 particles per frame. NIST (3125A Lot3125-004, 3200A Lot 3200-009, Thermo Scientific 3000 Series) standards were analyzed prior to sample analysis. The system was cleaned after each sample and checked for any sample carryover using the PBS diluent.

### Transmission Electron Microscopy (TEM)

Transmission electron microscopy for the analysis of whole mounted pEV samples was carried out as previously described, (14) at the Cell Imaging Facility at the University of Alberta Cross Cancer Institute. EVs were visualised using a Hitachi H-7650 Transmission Electron Microscope.

### Western Blots

pEV proteins were separated by electrophoresis using polyacrylamide gels (7% or 17%, depending on the molecular weight of target proteins) and transferred to polyvinylidene fluoride (PVDF) membranes. The blots were blocked with 5% non-fat dry milk and incubated overnight with the following primary antibodies from Thermo Fisher Scientific (CD9 (SN4 C3-3A2), CD63 (Ts63), CD81 (M38), and antibodies from abcam such as Apolipoprotein A1 (EPSISR27), Anti-HIV1 p55 + p24 + p17 antibody (ab63917), PD-1 (7A11B1), PD-L1 (AB_10986627) and TSG101 (4A10). Antibodies used at 1:500 dilution for CD9, CD81 and CD63 but at 1:1000 dilution for PDL-1 and PD-1. Membranes were incubated with HRP-conjugated secondary antibodies and developed using the enhanced chemiluminescence (ECL) detection kit (Thermo Fisher Scientific). Membranes were re-probed with loading controls (β-actin, GAPDH or tubulin). Protein bands of interest were quantified using Image Lab Software v6.0.1 (Bio-Rad).

### PBMCs-EV Co-Culture

PBMCs were treated with pEVs (100 µg, 200 µg or 400 µg; equivalent to ~ 1 x10^9^ -2 x 10^10^ pEVs) and stimulated with Staphylococcal enterotoxin B (SEB, 100 ng/ml) for 72hrs. The concentrations of IFN-γ and IL-2 in cell culture supernatants were determined by ELISA (R&D Systems). Unstimulated and untreated cells were used as negative controls.

### T Cell-EV Co-Culture

Total T cells were isolated using the EasySep™ Human T Cell Isolation Kit (STEMCELL Technologies) according to manufacturer’s instructions. Autologous PBMCs were treated with mitomycin C (Sigma Aldrich, 25 μg/ml) for 30min at 37°C, washed and plated with SEB-stimulated T cells at a ratio of 1:2. For some experiments, isolated T cells were labelled with CFSE (Molecular Probes) as previously described (15).

### EV Uptake Assay

To demonstrate the uptake of pEVs by PBMCs, primary T cells or Jurkat T cells, 5 x 10^9^ pEVs were labelled with CFSE (40 µM) for 2hrs at 37°C as described in (16). Labelled pEVs were applied to the centre of the gel bed at the top of Exo-Spin columns (Invitrogen) and spun at 750 x g for 2mins at room temperature to remove unbound dye. Cells were treated with labelled pEVs overnight followed by staining for Image flow cytometry. Data were analysed using the IDEAS software.

### Th1 Polarisation

Naïve CD4^+^ T cells were isolated using the EasySep Human Naïve CD4^+^ T cell isolation kit (STEMCELL Technologies). Isolated naïve CD4^+^ T cells were plated with pEVs (400 µg) and polarised for 5 days using the Human Th1 Cell Differentiation Kit (R&D Systems) according to manufacturer’s instructions.

### HIV Infectivity Assay

CD4^+^ T cells were isolated by negative selection using the EasySep Human CD4^+^ T cell enrichment kit (STEMCELL Technologies). The purity of isolated CD4^+^ T cells was >95%. CD4^+^ T cells were resuspended in RPMI media supplemented with 10% FBS, IL-2 (50 IU/ml) and activated with phytohemagglutinin (5 μg/ml, PHA) at a density of 2 x 10^6^ cells/ml. EVs (100 μg, 200 μg or 400 μg), isolated from the plasma of HC and HIV patients were added to CD4^+^ T cell cultures after 24hrs. Activated CD4^+^ T cells were infected with the CXCR4-utilising HIV-1 isolate, LAI, 24 hrs later, as previously described (17). Uninfected and untreated CD4+ T cells were used as controls. CD4^+^ T cells were harvested after 72-96 hrs, washed and stained for flow cytometry. Viral replication was analysed by intracellular p24 staining according to our previous reports (18, 19). For some experiments, unstimulated CD4^+^ T cells were pre-incubated with EVs for 24 hrs before HIV infection. For some experiments, EVs (HIV and HC, 100 µg) were labelled with live/dead fixable violet (Life Technologies) at a ratio of 1:100 for 2hrs at 37°C. Exo-Spin columns were used to remove unbound dye according to manufacturer’s instructions. Next, stimulated CD4^+^ T cells were treated with labelled EVs and infected the following day with the HIV-1 CCR5-tropic GFP-marked virus as previously described (17). Alternatively, aliquots of the CCR5-tropic GFP-marked virus were treated with an equal volume of labelled EVs overnight at 4°C and used to infect stimulated CD4^+^ T cells. Infected CD4^+^ T cells were harvested after 72hrs, stained and analysed for EV and virus uptake and EV-virus co-localisation by image flow cytometry.

### Viral Activation Assay Using J-Lat 10.6 Cells

J Lat 10.6 Jurkat cells obtained from NIH (AIDS reagents) which contain a full-length integrated HIV-1 genome that expresses GFP upon activation were treated with EVs (200 µg, HC, naïve or ART) for 72hrs. For some experiments, J Lat cells were treated with pEVs overnight followed by stimulation with soluble anti-CD3 (3 µg/ml) and anti-CD28 (1 µg/ml) for 72 hrs. Cells were harvested, washed and the expression of GFP and activation markers assessed by flow cytometry. For some experiments, unstimulated J Lat 10.6 Jurkat cells were treated with EVs and analysed after 72hrs by flow cytometry.

### Flow Cytometry

The following flow cytometry antibodies were obtained from BD Biosciences and Thermo Fisher Scientific: Anti-CD3 (SK7), anti-CD4 (RPA-T4), anti-CD8 (RPA-T8), anti-PD-1 (MIH4), anti-PD-L1 (MIH1), anti-TIGIT (MBSA43), anti-Ki67 (20Raj1), anti-CD38 (HIT2) and anti-HLA-DR (LN3). Live/Dead fixable Aqua (Life Technologies) was used for dead cell discrimination. For flow cytometry of EVs, 1μl aldehyde/sulphate latex beads (Thermo Fisher Scientific) were incubated with 4μl pEVs in 1ml double-filtered (0.2μm) PBS overnight at room temperature. The pEV-bead conjugates were blocked with glycine for 30mins, centrifuged at 5000 rpm for 5mins, washed twice with 1ml PBS supplemented with 0.5% Exo-free FBS and labelled with the following antibodies (BioLegend, anti-CD9 (HI9a), anti-CD63 (Ts63, Thermo Fisher), anti-CD81 (1D6-CD81, Thermo Fisher), anti-TSG101 (EPR7130B, abcam), anti-Flotillin1 (EPR6041, abcam), anti-TCRαβ (T10B9.1A-31, BD Biosciences), anti-HLA-G (BD Biosciences), anti-CCR5 (2D7, BD Biosciences), anti-CXCR4 (12G5, BD Biosciences), anti-PD-1 (MIH4, Thermo Fisher) and anti-PD-L1 (MIH1, Thermo Fisher). Intracellular cytokine staining for IFN-γ, T-bet, IL-17 and IL-4 was performed according to our previous reports (20, 21). Data was acquired using a BD Fortessa flow cytometer followed by analysis using FlowJo v10.6 software.

### PD-L1 ELISA

The concentrations of PD-L1 in whole plasma, EV-depleted plasma and pEVs (200 μg) were determined by ELISA (Invitrogen) according to manufacturer’s instructions.

### RNA Isolation and RT-PCR

Total RNA was isolated from primary T cells or Jurkat cells using the RNeasy Mini Kit (Qiagen). 100 ng-200 ng reverse transcription reactions for cDNA synthesis were carried out using the miScript RT Kit (Qiagen) according to the protocol for subsequent quantification of miRNAs and mRNAs.

Real-time PCR (RT-PCR) analysis of gene (mRNA) and miRNA expression was carried out using Quantitect SYBR Green Primer Assays (Qiagen, for mRNAs) and miScript SYBR Green Primer Assays (Qiagen, for miRNAs) and the Bio- Rad CFX96 real-time cycler. For some gene expression analysis, we used the RT^2^ qPCR Primer Assays (Qiagen). βeta-2 microglobulin (β2M) was used as an internal control for gene expression. The small nuclear RNA, U6, was used as an internal control for miRNA expression. For extravesicular miRNA expression, C*. elegans* miR-39 which detects the Spike-In Control added to samples during EV RNA isolation was used for normalization of RT-PCR results for miRNA expression. For data analysis, we used the ΔΔC_T_ method of relative quantification.

### Human T and B Cell Activation miRNA PCR Array

A miRNA PCR array consisting of 84 miRNAs associated with human T and B cell activation (Qiagen) was used to identify miRNAs differentially expressed by pEVs from HIV patients [naïve (12), ART (12)] compared to healthy controls. 5 µl of EV RNA was reverse transcribed using the miScript RT Kit (Qiagen) reverse transcription protocol for subsequent quantification of mature miRNAs according to manufacturer’s instructions. The reverse transcription reaction was diluted with RNAse-free water according to manufacturer’s specifications followed by RT-PCR for mature miRNA profiling using the Quantitect SYBR Green Kit. For data analysis, we used the ΔΔC_T_ method of relative quantification for miScript miRNA PCR Arrays indicated at http://pcrdataanalysis.sabiociences.com/mirna. For data normalisation, we used the global C_T_ mean of expressed miRNAs method which automatically calculates a global C_T_ mean for the miRNA targets that are commonly expressed in all samples being analyzed. C_T_ values equal to or greater than 35 were regarded as negative calls.

### PD-1/PD-L1 Cell-Based Assay

Recombinant Jurkat T cells which constitutively express PD-1 (BPS Bioscience, CA, USA) and Chinese Hamster Ovary (CHO) cells which constitutively express PD-L1 and an engineered TCR activator were purchased from BPS Bioscience (CA, USA). PD-1+ Jurkat cells were pre-incubated with pEVs (HC, ART-naïve or ART) and co-cultured with CHO cells according to manufacturer’s instructions and our previous studies (22, 23). Briefly, 2.5 x 10^5^ Jurkat cells were pre-incubated with 100 µg pEVs overnight followed by co-culture with 4 x10^4^ CHO cells per well in the presence of anti-PD-L1 monoclonal antibody (10 µg/ml) for 6hrs at 37°C.

### pEV Transfection

pEVs were transfected with miR-139-5p inhibitor (500 nM) using the Exo-fect™ siRNA/miRNA transfection kit (System Biosciences) according to manufacturer’s instructions. Transfected pEVs (HC, HIV naïve and ART) were applied to J-Lat 10.6 cells. Cells were washed after overnight incubation, fresh media added and harvested after 72hrs. Mock transfected pEVs were used as controls.

### Statistical Analyses

Statistical analyses were performed using Graph Pad Prism v.8.0. Normality of distribution was determined by the Kolmogorov-Smirnov normality test. For normally distributed data, statistical significance was determined using the unpaired Student’s t test with Welch’s correction. The Mann-Whitney non-parametric test was used for unpaired data that were not normally distributed. The Wilcoxon matched-pairs test was used for paired data that are not normally distributed. Ordinary one-way ANOVA was used to analyse more than two groups, while 2-way ANOVA was applied for analyses of two or more groups with multiple parameters. The Dunnett’s, Tukey’s and Sidak’s multiple comparisons tests were used to test simple effects between groups. Pearson’s Correlation was used for correlation analysis, the P-values less than 0.05 were considered statistically significant.

## Results

### Characterisation and Size Determination of pEVs From HIV Patients and HCs

**Figure 1:**
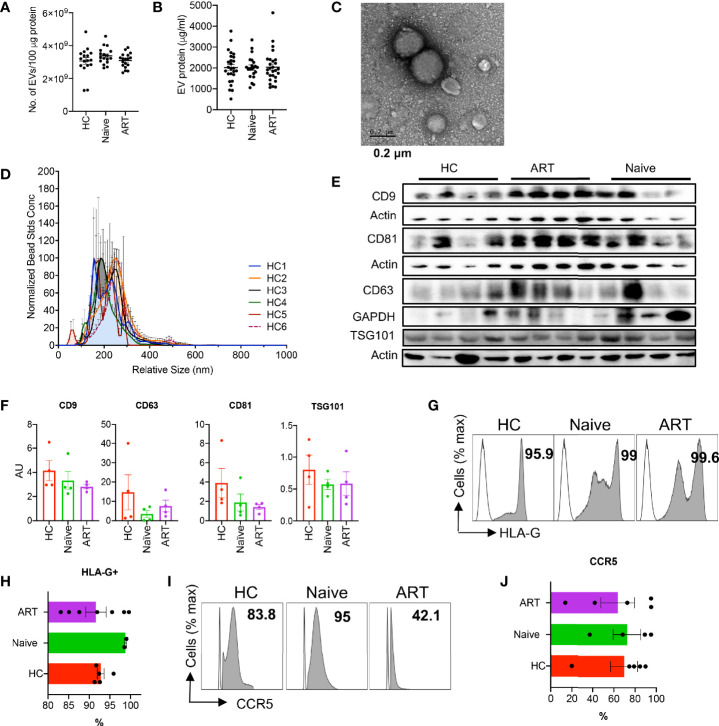
Characterisation of plasma EVs. **(A)** Number of EVs isolated from plasma samples of HCs, HIV ART-naïve and HIV patients on ART. Error bars indicate the mean ± SEM of pEV samples analysed from 3 independent experiments. **(B)** pEV (HC, HIV ART-naïve and HIV on ART) protein concentrations obtained from BCA assay. Error bars indicate the mean ± SEM of pEV samples analysed from 3 independent experiments. **(C)** Representative TEM image of pEVs. Scale bar, 0.2 μm. **(D)** Analysis of pEV size by Nanoparticle Tracking Analysis (NTA) and merged histograms depicting the size (nm) of 6 HC pEV samples shown. **(E)** Representative immunoblots of band intensities of CD9, CD63, CD81and TSG101 in isolated pEVs from HCs, ART-naïve and ART patients normalised to the loading control (Actin or GAPDH). All blots were repeated twice for reproducibility and lanes were loaded with equal volumes of pEV samples. **(F)** Quantification of EV CD9, CD63, CD81 and TSG101 normalised to the loading control. Error bars indicate mean± SEM of 4 samples per group from two independent experiments. **(G)** Representative images (open plots are isotype controls and filled grey plots are showing HLA-G) and **(H)** cumulative data of HLA-G expressed by HC, ART-naïve and ART pEVs. Data from five (HC and ART) and two pEV samples shown. **(I)** Representative histogram plots (open plots are isotype controls and filled grey plots are showing CCR5), and **(J)** Accumulative expression of CCR5 by HC, ART-naïve and ART pEVs. Data from five (HC and ART) and two pEV samples shown. Error bars indicate mean ± SEM.

We isolated pEVs from 1-4 ml of EDTA plasma samples by precipitation or by using affinity spin columns (24). Although studies have shown that HIV patients have higher numbers of pEVs than HCs (11, 25, 26) we did not observe any difference in the concentrations of EV protein and numbers obtained from the plasma of HCs, HIV ART-naïve and HIV patients on ART (**Figures 1A, B** **)**. pEV yields ranged from 1 x 10^9^ – 4 x10^9^ pEVs/100 µg protein (**Figure 1A**).

Next, we verified the size of isolated pEVs by transmission electron microscopy (TEM, **Figure 1C**) and nanoparticle tracking analysis (NTA, **Figure 1D**). Isolated pEVs consisted of both small and large vesicles, with sizes ranging from 55 nm to 300 nm (**Figure 1D** and **Supplementary Figures 1A–F**). pEVs are characterised based on their cargoes which comprise of various proteins, lipids and nucleic acids (5). We found that pEVs isolated from HCs and HIV patients harbour varying levels of tetraspanins (CD9, CD63, CD81), flotillin-1 and TSG101, (**Figures 1E, F** and **Supplementary Figures 1G, H** **)**. In addition, we detected apolipoprotein A (ApoA), indicative of the precipitation and affinity spin column methods of EV isolation (**Supplementary Figures 1I, J**) (27–29). Importantly, isolated pEVs did not harbour the negative control marker, cytochrome C (**Supplementary Figure 1K**).

Moreover, we found that pEVs harbour various cell type-specific proteins including high levels of HLA-G (**Figures 1G, H** **)**, indicative of their plasma origin (30). pEVs from all groups expressed varying levels of markers associated with dendritic cells and T cells such as HLA-DR and TCRαß (**Supplementary Figures 1L, M** **)**. We also found that pEVs express high levels of the HIV co-receptor CCR5 (**Figures 1I, J** **)**, but low levels of CXCR4 (**Supplementary Figures 1L, M** **)**. Collectively, these results indicate that pEVs are a heterogeneous population of vesicles which express varying levels of tetraspanins and other markers possibly associated with cellular origin and pathological state (5).

### Increased Inflammatory Responses by PBMCs and T Cells Treated With HIV+ ART- Naïve pEVs

**Figure 2:**
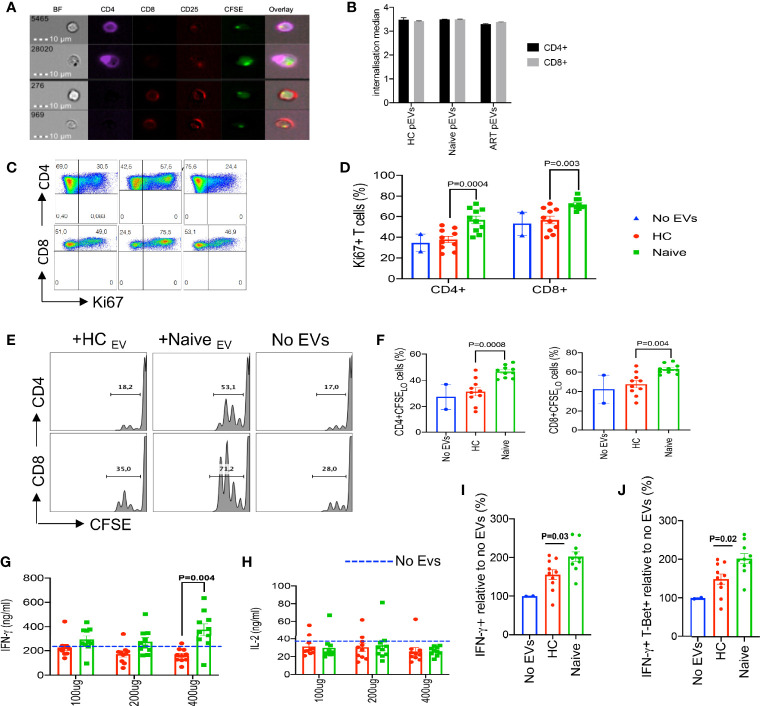
Increased inflammatory responses by PBMCs and T cells treated with HIV ART-naïve pEVs. **(A)** Representative images of stimulated CD4^+^ and CD8^+^ T cells incubated with CFSE labelled pEVs. **(B)** Internalisation score of HC, ART-naïve and ART pEV uptake by CD4^+^ and CD8^+^ T cells. **(C)** Representative flow cytometry plots, and **(D)** cumulative data of the frequency of ki67 expressing CD4^+^ and CD8^+^ T cells left untreated (No EVs) or pre-incubated with pEVs from HC or HIV ART-naïve patients. **(E)** Representative flow plots, and **(F)** cumulative data of the proliferation of stimulated CD4^+^ and CD8^+^ T cells left untreated (No EVs) or pre-incubated with pEVs from HCs or HIV ART-naïve patients, indicated by CFSE dilution. **(G)** Concentrations of IFN-γ produced by PBMCs pre-incubated with pEVs (100 μg, 200 μg or 400 μg) from HCs or HIV ART-naïve patients before stimulation with SEB (100 ng/ml) for 72hrs. Dotted lines indicate the levels of IFN-γ produced by untreated PBMCs. **(H)** Concentrations of IL-2 produced by PBMCs pre-incubated with pEVs (100 μg, 200 μg or 400 μg) from HCs or HIV ART-naïve patients. **(I)** Fold percentage of IFN-γ expression, and **(J)** co-expression of IFN-γ and T-Bet+ by naïve CD4^+^ T cells pre-incubated with pEVs from HCs or HIV ART-naïve patients and stimulated under Th1-polarising conditions. Representative data from 3 independent experiments shown. Data points within pEV groups represent individual pEV samples. Error bars indicate mean±SEM. Indicated p-values were obtained from the Dunnett’s multiple comparisons test.

As intercellular communicators, EVs can transfer proteins and genetic material that can modulate the function of recipient cells. Similarly, we found that CD4^+^ and CD8^+^ T cells can internalise CFSE-labelled pEVs after overnight incubation (**Figures 2A, B** **)**. Studies have indicated that pEVs from HIV patients harbour significantly higher levels of inflammatory effectors such as ADAM17, TNF-α and IL-6 compared to HCs. To determine whether pEVs from HIV+ ART-naïve patients were more inflammatory than pEVs from HCs and can hence differentially modify T cell function, we pre-incubated PBMCs or isolated T cells with pEVs overnight before stimulation. As shown in **Figures 2C, D**, we found that CD4^+^ and CD8^+^ T cells pre-incubated with pEVs from HIV+ ART-naïve patients were more activated (based on Ki67 expression) and had more proliferative capacity compared to cells pre-incubated with pEVs from HCs and untreated controls (**Figures 2E, F** **)**. Furthermore, we found that PBMCs pre-incubated with pEVs from HIV+ ART-naïve patients elicited higher levels of IFN-γ than pEVs from HCs, particularly when higher concentrations of pEVs were used (400 μg pEV protein, **Figure 2G**). However, pre-incubation of PBMCs with HIV+ ART-naïve and HC pEVs, did not impact IL-2 production as similar levels of IL-2 were produced by pre-incubated and untreated cells (**Figure 2H**).

Based on our observation that HIV+ ART-naïve pEV-treated PBMCs produced higher amounts of IFN-γ (**Figure 2G**), we hypothesised that pre-treating naïve CD4^+^ T cells with pEVs may increase their polarisation to Th1 cells. As expected, pre-incubation with ART-naïve pEVs from HIV+ individuals induced a 2-fold increase in the frequency of Th1 polarised cells (IFN-γ+ and IFN-γ+T-Bet+ cells) compared to untreated cells (**Figures 2I, J** **)**. Furthermore, we observed that ART-naïve pEVs elicited the polarisation of higher frequencies of Th1 cells, compared to HC pEVs (p=0.03, IFN-γ+ cells, p=0.02, IFN-γ+T-Bet+ cells, respectively, **Figures 2I, J** **)**. Although HC pEVs also induced increased Th1 polarisation in comparison to untreated naïve CD4^+^ cells, it did not reach a significant level (**Figures 2I, J** **)**. These results demonstrate that pEV uptake can enhance the stimulation of recipient T cells. In addition, our results indicate that EVs isolated from HIV+ART-naïve plasma harbour more inflammatory mediators in comparison to EVs isolated from the plasma of HCs.

### pEVs From HCs and HIV Patients Enhance HIV Infection of Activated CD4^+^ T Cells

**Figure 3:**
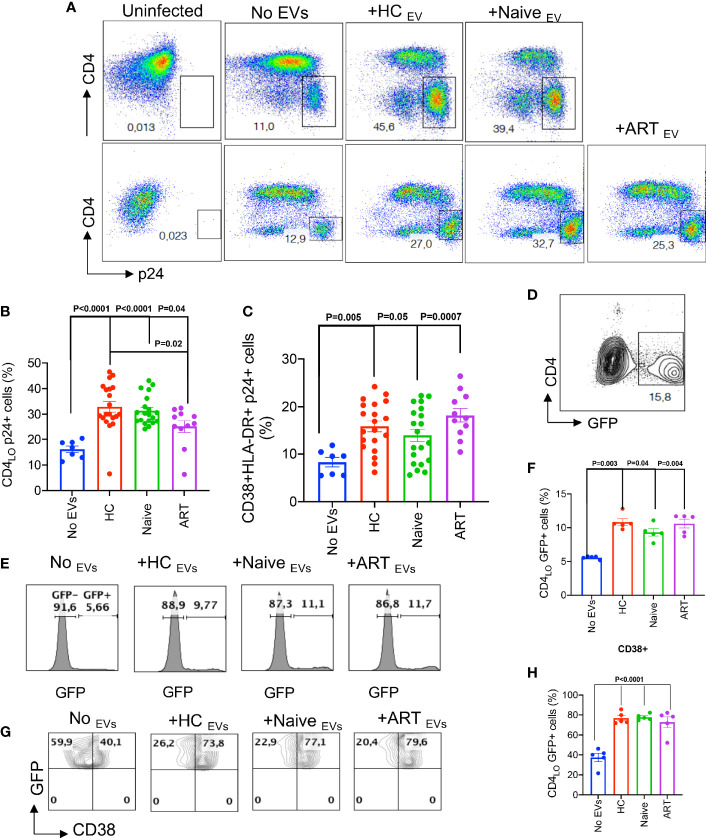
pEVs from HCs and HIV patients enhance HIV infection of activated CD4^+^ T cells. **(A)** Representative flow plots of infected CD4+ T cells (p24+) pre-incubated with the indicated pEVs. Results (upper and lower panels) from two experiments shown. p24 gates were derived from the uninfected plots shown. **(B)** Cumulative frequencies of p24 expressed by activated CD4^+^ T cells untreated (No pEVs) or pre-incubated with the indicated pEVs and infected with HIV-1 LAI virus. **(C)** Cumulative data of the frequencies of CD38 and HLA-DR co-expressed by p24+ cells in activated CD4^+^ T cells pre-incubated with the indicated pEVs. **(D)**, Representative plot of GFP expressing CD4^+^ T cells. **(E),** Representative histograms of GFP expression by J-Lat 10.6 CD4^+^ T cells and **(F)**, cumulative frequencies of GFP expression by CD4^+^ T cells in response to incubation with the indicated pEVs. **(G)**, Representative contour plots showing expression of CD38+GFP+ on gated CD4 cells and **(H)**, cumulative frequencies of CD38 expression by CD4 GFP+ cells in response to incubation with the indicated pEVs. Quadrants were derived from plots of untreated cells shown. Data points within pEV groups represent individual pEV samples. Representative data from 3 independent experiments shown. Error bars indicate mean ± SEM. p-values were obtained from the Dunnett’s multiple comparisons test.

Based on these observations on the stimulatory properties of pEVs, we decided to investigate whether infection of activated CD4^+^ T cells with HIV could be enhanced by pEV pre-incubation. To achieve this, we isolated CD4^+^ T cells (**Supplementary Figure 2A**), then pre-incubated activated CD4^+^ T cells with pEVs from HCs, HIV infected on ART or ART-naïve individuals before infection with the CXCR4-tropic HIV LAI virus. We found that the percentage of infected CD4^+^ T cells pre-incubated with pEVs was significantly higher than untreated cells (**Figures 3A, B** **)**. Interestingly, the infection rates mediated by pEVs from HCs were comparable to HIV ART-naïve pEVs, albeit lower in ART pEV-treated CD4^+^ T cells (HCs *vs* ART, p=0.02, **Figures 3A, B** **)**. As expected, the cumulative percentage of activation markers expressed by pEV-treated CD4^+^ T cells was higher than untreated cells, which corresponded with the frequency of infected cells (CD38+HLA-DR+, **Figure 3C**).

We also investigated whether pEVs could reactivate latent HIV-infected cells. We found that treating unstimulated latently infected J-Lat10.6 cells with pEVs, irrespective of the source, resulted in significantly higher GFP expression, indicative of viral reactivation, compared to untreated cells (**Figures 3D–H**). We also observed that pEV treatment increased cell activation, based on CD38 expression (p=<0.0001, **Figures 3G, H** **)**. We obtained similar results when J-Lat10.6 cells were pre-incubated with pEVs before stimulation with anti-CD3 antibody (**Supplementary Figure 2B**). These results confirm that pEVs can promote viral re-activation as well as infectivity.

We also evaluated the effect of timing on pEV-mediated CD4^+^ T cell activation and HIV infection. We found that unlike pre-incubation, pEV treatment post-infection did not impact the frequency of p24+ CD4^+^ T cells (**Supplementary Figure 2C**). These results indicate that pEVs *prime* or *educate* CD4^+^ T cells, resulting in increased T cell activation and HIV infection. We also investigated whether pEVs can promote HIV infection of resting CD4^+^ T cells. However, we noted that the percentage of HIV-infected p24+ cells were very low with or without pEV treatment (**Supplementary Figures 2D, E** **)**.

### Prior Virus Interaction Distinguishes the Ability of pEVs From HIV ART-Naïve Patients to Mediate Increased HIV Infectivity Compared to pEVs From HCs

**Figure 4:**
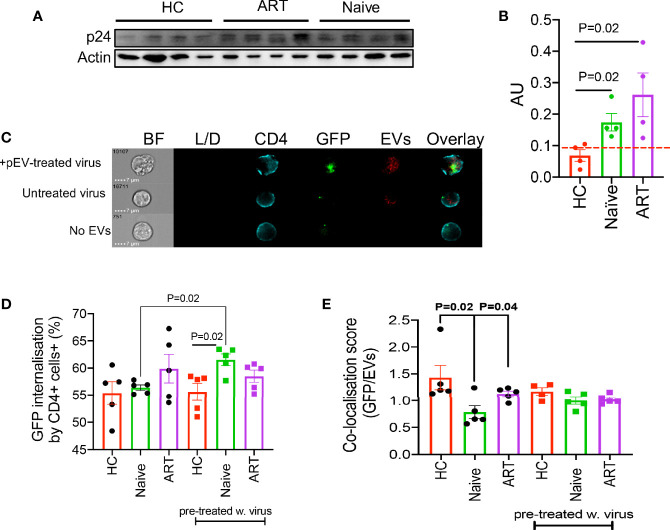
**(A)** Representative blot of p24 and actin by indicated pEVs (4 per group). **(B)** Cumulative expression of p24 by the indicated pEVs relative to actin. Data from two experiments shown. Pre-incubation of HIV-1 CCR5-tropic GFP-virus with HIV naïve pEVs increases virus infectivity. **(C)** Representative images showing intensity of GFP and violet-labelled pEVs in CD4^+^ T cells infected with pre-treated virus (top), CD4^+^ T cells pre-incubated with pEVs followed by infection with untreated virus (middle) and CD4^+^ T cells infected with virus in the absence of pEVs (bottom). **(D)** Percentage of virus (GFP) internalisation by CD4^+^ T cells infected with CCR5-tropic GFP virus followed by pEV treatment (HCs, HIV ART-naive and patients on ART) or CD4^+^ T cells infected with pEV pre-incubated CCR5-tropic GFP virus (pre-treated w.virus). **(E)** Co-localisation score of GFP (virus) and violet-labelled pEVs in CD4+ T cells infected as described in **(D)**. Data points represent individual samples within pEV groups. Error bars indicate mean±SEM. Representative data from 3 independent experiments shown. Indicated p-values were obtained from the Welch’s t test **(B)**, Paired t test **(D)** and the Dunnett’s multiple-comparison test **(E)**. Error bars indicate mean±SEM. Indicated p-values obtained from the Dunnett’s multiple comparisons test.

EVs secreted by HIV-infected cells harbour proteins or receptors associated with virus entry such as Nef, Gag, CXCR4 and CCR5 due to the overlap between biogenesis and the production of virions (31, 32). We found that ART-naïve and ART pEVs harboured significantly higher levels of Gag (p24) protein than background (p=0.02 compared to HC pEVs, **Figures 4A, B** **)**. Surprisingly, we observed a faint response to the anti-p24 antibody in HCs even when we tested a different clone of the anti-p24 antibody. Consequently, we investigated whether pre-exposing virus to pEVs could impact the infection rate of CD4^+^ T cells. As pEVs harboured higher levels of the CCR5 than CXCR4, we pre-incubated the CCR5-tropic virus with pEVs from HIV-naïve, HIV ART and HCs. We observed that pre-exposure of the GFP-marked CCR5-tropic virus to pEVs from HIV ART-naïve patients corresponded with significantly higher frequencies of GFP expression by infected CD4^+^ T cells compared to HC pEVs (p=0.02, **Figures 4C, D**, (pre-treated w. virus)). Although pre-exposing the virus to pEVs from HIV patients on ART resulted in a higher HIV-infection by recipient CD4^+^ T cells, it did not reach a significant level [**Figure 4D**, (pre-treated w. virus)]. On the other hand, the frequencies of GFP expressed by CD4^+^ T cells pre-incubated with pEVs and infected with the GFP-marked CCR5-tropic virus 24 hrs later, were similar between the groups (**Figure 4D**). Interestingly, we found pre-exposure of the GFP-virus with HIV ART-naïve pEVs, but not other groups yielded significantly higher infected CD4^+^ T cells than untreated virus (p=0.02, **Figure 4D**). The increased GFP-virus internalisation by CD4^+^ T cells pre-exposed to HIV ART-naïve pEVs is an indication that pEVs isolated from HIV ART-naïve samples may harbour factors which enhance viral uptake by CD4^+^ T cells. Consequently, we assessed virus (GFP)/pEV co-localisation in CD4^+^ T cells infected with virus, which had been pre-exposed to pEVs or just pre-incubated with pEVs before infection. The co-localisation scores (GFP/pEVs) were similar for CD4^+^ T cells infected with virus pre-exposed to pEVs from all groups analysed [**Figure 4E**, (pre-treated w. virus)]. However, we found that the co-localisation scores for CD4^+^ T cells pre-incubated with pEVs from HCs and HIV ART patients and infected with virus 24hrs later were significantly higher than cells pre-incubated with ART-naïve pEVs (p=0.02, P=0.04 respectively, **Figure 4E**). Taken together, these results indicate that pEVs secreted by cells exposed to HIV harbour factors which promote the infection of healthy CD4^+^ T cells. Furthermore, our results demonstrate the ability of HIV to co-localise with pEVs. The lower co-localisation score of CD4^+^ T cells pre-incubated with pEVs from HIV ART-naïve patients, compared to HCs and HIV ART patients may signify the downregulation or reduced expression of the HIV co-receptor, CCR5 (33).

### pEVs From HIV Patients and HCs Express PD-1 and PD-L1

**Figure 5:**
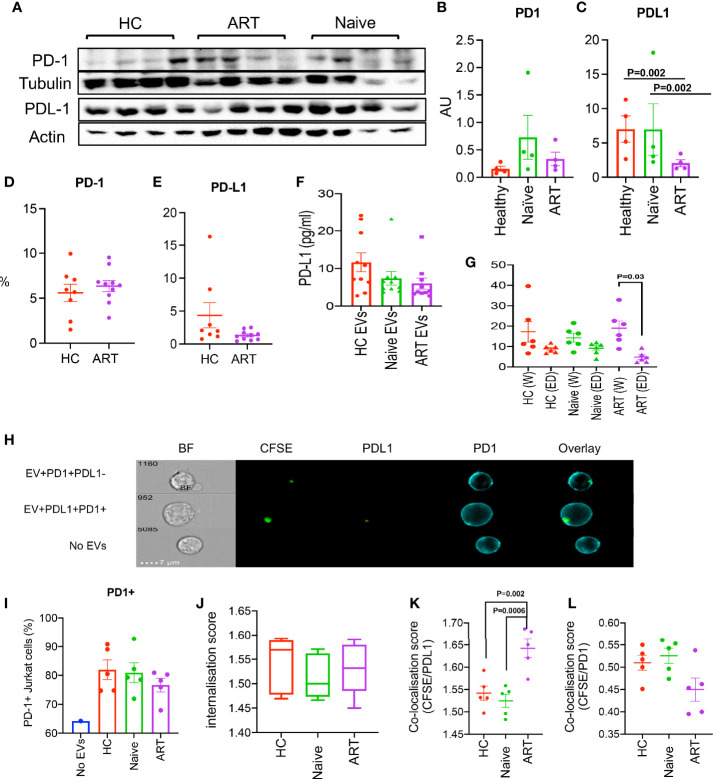
**(A)** Representative immunoblots of PD-1 and PD-L1 in isolated pEVs from HCs, HIV ART-naïve and HIV patients on ART (4 samples per group). All blots were repeated for reproducibility and lanes were loaded with equal volumes of pEVs. **(B, C)** Quantification of PD1 and PD-L1 indicated in **(A)** normalised to the loading control. Error bars indicate mean ± SEM of 4 samples per group from two independent experiments. **(D, E)** Cumulative flow cytometry data of the percentages of PD1 and PD-L1 in pEVs from HCs and HIV patients on ART. **(F)** Data showing concentrations of PD-L1 in pEVs from HCs, HIV ART-naïve and HIV patients on ART. **(G)** Concentrations of PD-L1 in whole (W) and EV-depleted (ED) plasma from the indicated groups. Data from 3 experiments shown. p-value (p=0.03) was obtained from the Wilcoxon matched-pairs signed rank test **(G)**. Error bars indicate mean ± SEM. p-values were obtained from the Tukey’s multiple-comparison test **(C)**. **(H)** Representative images of PD-1+ Jurkat cells stimulated with soluble anti-CD3/CD28 overnight and incubated with CFSE-labelled pEVs for 3hrs. Jurkat cells were stained with anti-PD-1 and anti-PD-L1 antibodies. Top plot, CFSE (pEV)+PD-1+PD-L1- cells, middle plot, (pEV)+PD-1+PD-L1+ cells, bottom plot, no pEVs. **(I)** Percentages of PD1+ Jurkat cells untreated or treated with the indicated pEVs o/n (5 per group). **(J)** CFSE internalisation scores of Jurkat cells pre-incubated with the indicated pEVs. **(K)** Co-localisation scores of CFSE (pEVs) and PD-L1 or **(L)** PD-1 of Jurkat cells pre-incubated with the indicated pEVs. Error bars indicate mean±SEM. p-values were obtained from the Tukey’s multiple comparisons test **(K)**. Data from 3 experiments shown.

In chronic conditions, CD4^+^ and CD8^+^ T cells express various co-inhibitory receptors such as PD-1 and CTLA-4, to counter excessive inflammatory responses and immunopathology (34). It is reported that tumour cells release EVs which harbour PD-L1 and thus promote immunosuppression and the development of pre-metastatic niches (35, 36). The co-expression of PD-1 and other co-inhibitory receptors such as TIM-3, Gal-9, LAG-3, 2B4, TIGIT and CD160 by HIV-specific CD8^+^ T cells are hallmarks of HIV severity and T cell dysfunction (20, 21, 37–39). Hence, it is likely that pEVs secreted by cells from HIV patients harbour high levels of co-inhibitory receptors and ligands, which may modulate recipient T cell responses. Therefore, as a proof-of-concept, we examined whether pEVs from HIV-naïve, on-ART patients and HCs harboured PD-L1 and its receptor, PD-1. We detected slightly higher levels of PD-1 protein in pEVs isolated from HIV ART and ART-naïve patients in comparison to HCs, however this was not significant (**Figures 5A, B** **)** but the levels of PD-L1 protein in pEVs was higher compared to PD-1 (**Figures 5A, C** **)**. Interestingly, pEVs isolated from HCs and HIV ART-naïve patients harboured higher levels of PDL-1 protein compared to pEVs from ART patients (p=0.002, **Figure 5C**).

We also assessed the surface expression PD-1 and PD-L1 by flow cytometry (using EV-labelled aldehyde/sulphate latex beads, **Figures 5D, E** **)**. We detected similar levels of PD-1 in pEVs from HCs and HIV ART patients; however, the surface expression of PD-L1 detected by this method was very low (**Figure 5E**). These results may be indicative of the differences between the levels of pEV proteins detected by western blotting and flow cytometry. Nevertheless, our results show that pEVs from HIV patients and HCs harbour varying levels of PD1 and PD-L1, which may be synonymous with the phenotypes of their parent cells.

As we detected high levels of PD-L1 protein in pEVs by western blotting compared to PD1, we decided to measure the concentration of extravesicular PD-L1 by ELISA. PD-L1 levels detected from pEVs from HCs, HIV ART-naïve and ART patients were comparable and ranged from 5-25 pg/ml (**Figure 5F**). Next, we investigated whether pEVs are the main source of PD-L1 in the plasma from HC and HIV patients (ART-naïve and ART). We found that the concentration of PD-L1 in whole plasma from ART patients was significantly higher than EV-depleted plasma (p=0.03, **Figure 5G**). We also observed a similar trend between the whole and EV-depleted plasma from ART-naïve patients, although this was not significant (**Figure 5G**). These observations indicate that pEVs are a source of PD-L1 in HIV patients and HCs.

Next, to further investigate the expression of PD-L1+ by pEVs, we examined the uptake of CFSE-labelled pEVs by PD1+ Jurkat cells after overnight treatment (**Figure 5H**). PD-1+ Jurkat cells incubated with CFSE-labelled pEVs overnight were labelled with a-PD1 and a-PD-L1 antibodies and analysed by image cytometry. As expected, Jurkat cells were PD1+, however cells pre-incubated with pEVs expressed higher levels of PD1 than untreated cells. (**Figure 5I**). The CFSE internalisation scores of Jurkat cells pre-incubated with pEVs from all groups analysed were similar (mean~1.5, **Figure 5J**). The CFSE/PD-L1 co-localisation score of Jurkat cells pre-incubated with pEVs from HIV-patients on ART was significantly higher than cells treated with pEVs from HC and HIV ART-naïve patients (mean=1.64; p=0.002 and 0.0006 respectively, **Figure 5K**). On the other hand, the CFSE/PD-1 co-localisation score of Jurkat cells pre-incubated with pEVs from HIV patients on ART was lower than cells treated with pEVs from HC and HIV ART-naïve patients, however this was not significant (**Figure 5L**). These results indicate that pEVs from HC and HIV patients express both PD1 and PD-L1.

### Differential miRNA Expression by pEVs From HIV Patients Compared to HCs

**Figure 6:**
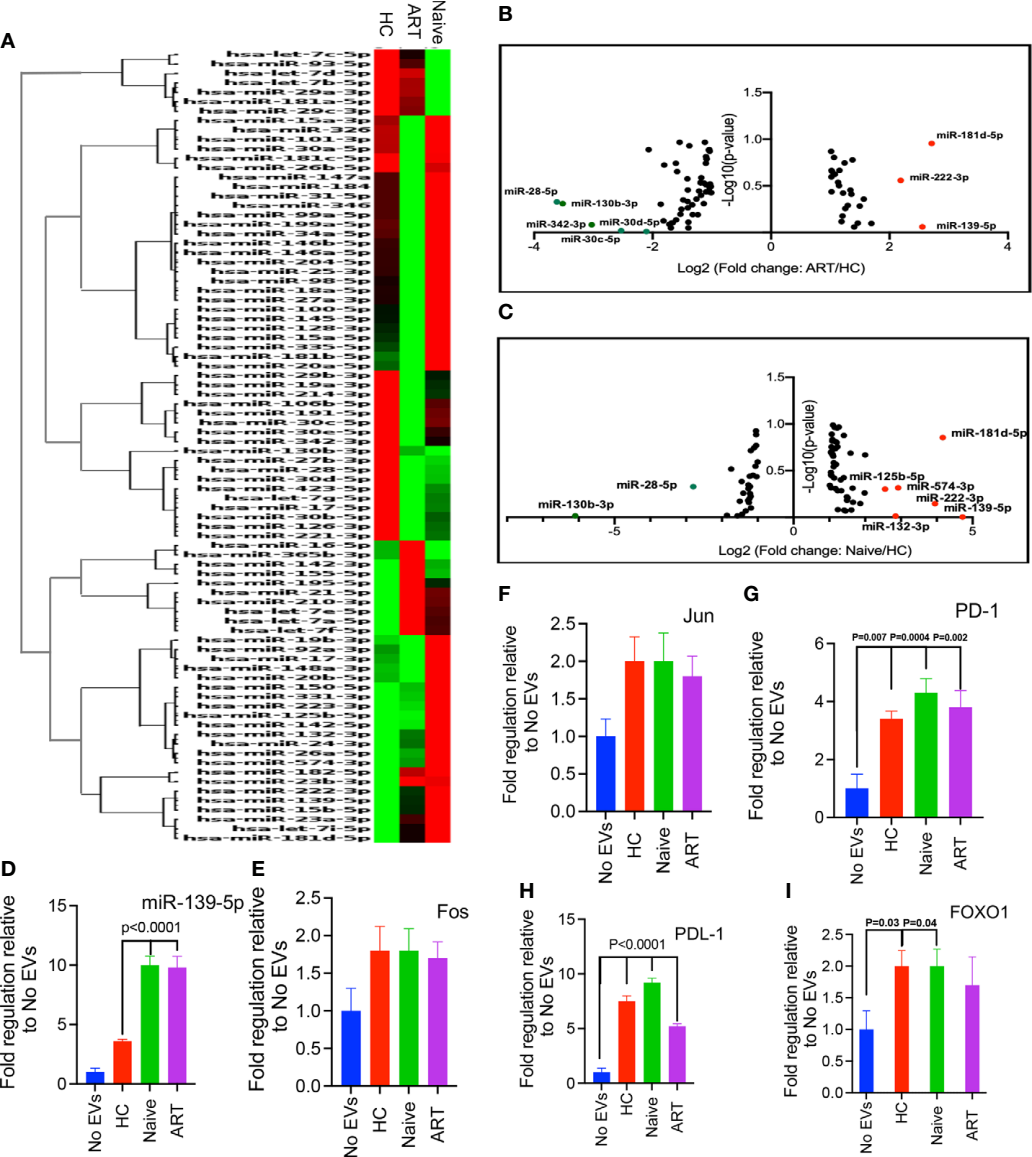
**(A)** Hierarchical clustering of miRNAs expressed by pEVs from HCs (H), HIV patients on ART (A), and HIV ART-naïve (N) patients. The heatmap lane for each group represents the mean of 12 biological replicates (H, A, N)**. (B, C)**. Volcano plots of log_10_ p-values versus log_2_ fold change, depicting miRNAs differentially expressed by pEVs isolated from HIV patients on ART and ART-naïve patients, compared to HC pEVs. Red dots, upregulated miRNAs (fold-change threshold of ≥2), green dots, downregulated miRNAs (fold-change threshold of ≤-2). **(D)** Fold expression of miR-139-5p by PD-1+ Jurkat cells pre-incubated with the indicated pEVs (4-6 pEV samples per group) overnight followed by stimulation with PD-L1+ CHO cells for 6hrs in the presence of anti-PD-L1 antibody (10 ug/ml). **(E–I)** Fold expression of Fos **(E)**, Jun **(F)**, PD1 **(G)**, PD-L1 **(H)** and FOXO1 **(I)** by stimulated PD-1+ Jurkat cells as described above. Error bars indicate mean±SEM. p-values were obtained from the Dunnett’s multiple comparison test.

EVs harbour various proteins, genetic information in the form of RNAs and extra-chromosomal DNA, which are mostly synonymous with their parent cells (6). To determine whether pEVs modulate T cell responses by miRNA transfer, we first evaluated whether pEVs isolated from HIV patients and HCs harboured miRNAs associated with T and B cell activation. From hierarchical clustering analysis, we observed the variability of miRNA harboured by pEVs from individual HCs, HIV ART and HIV ART-naive patients (**Supplementary Figure 3A**). However, clustering analysis based on average group miRNA expression indicate similarities and differences in miRNA expression which may be attributed to HIV infection or ART treatment (**Figure 6A**). miR-139-5p was upregulated in pEVs from HIV-infected on ART and ART-naïve patients although this was only significant in pEVs from ART-naïve patients (**Figures 6B, C** and **Supplementary Figure 3B**). Other miRNAs upregulated in pEVs from HIV ART and ART-naïve patients (≥2-fold regulation) compared to HC pEVs were: miR-132-3p, miR-125b-5p, miR-181d-5p, miR-222-3p and miR-574-3p (**Figures 6B, C** and **Supplementary Figure 3B**). miR-130b-3p, miR-30c-5p and miR-30d-5p were significantly downregulated in pEVs from HIV-infected ART-naïve and ART patients respectively (**Supplementary Figure 3C**). miR-342-3p and miR-26b-5p levels were also reduced, although not significantly (fold regulation ≥2-fold less in HIV ART and ART-naïve pEVs, **Supplementary Figure 3C**). Next, we validated the expression of miR-139-5p by HIV ART-naïve and ART pEVs relative to HCs by RT-PCR (same samples as array). In contrast to the PCR array results, we only observed a ~2-fold increase in miR-139-5p harboured by pEVs from HIV ART-naïve and ART patients in comparison to pEVs from HCs (**Supplementary Figure 3D**). We attribute the differences between the PCR array and validation RT-PCR results to the normalisation methods used as described in the Materials and Methods section.

As the levels of miR-139-5p harboured by pEVs from HIV ART-naïve and ART patients were approximately 2-fold higher than pEVs from HCs, we decided to investigate whether pEVs can modulate T cell responses by transferring this miRNA to recipient T cells.

### miR-139-5p Expression Is Upregulated in Stimulated PD-1+ Jurkat Cells Pre-Incubated With pEVs

Next, we decided to investigate whether pEVs can modulate T cell function through PD1 and PD-L1 signalling. To achieve this, we utilised a PD-1-PD-L1 cell-based assay based on the interaction between PD1+ Jurkat cells and PD-L1+ CHO cells expressing a TCR activator. Jurkat cells were pre-incubated with pEVs (HC, HIV ART and HIV ART-naïve) overnight. An antibody that targets PD-L1 was added to the assay to promote Jurkat cell activation. miRNAs modulate biological processes by binding to the 3’UTR of target mRNAs, thereby mediating translational repression or mRNA destabilisation (10). The components of the transcription factor Activator Protein 1 (AP-1): *c-Jun, JunB, c-Fos* and *BATF*; induce the expression of *PD-1* and *PD-L1* (40, 41). Furthermore, *c-Fos* (Fos) and *c-Jun* (Jun) have been identified as miR-139-5p targets by computational analysis and experimental studies (42, 43). Consequently, we evaluated the expression of miR-139-5p, *Fos* and *Jun* by PD-1+ Jurkat cells. miR-139-5p was significantly upregulated in response to pre-incubation with pEVs (p<0.0001, **Figure 6D**). Furthermore, Jurkat cells pre-incubated with ART-naïve and on ART pEVs expressed significantly higher levels of miR-139-5p compared to HC pEVs (p<0.0001, **Figure 6D**). PD1+ Jurkat cells pre-incubated with pEVs from all groups expressed higher levels of *Fos* and *Jun* compared to untreated cells, however these differences did not reach significance (**Figures 6E, F** **)**. We also evaluated *PD-1* and *PD-L1* expression by Jurkat cells. Jurkat cells pre-incubated with pEVs expressed significantly higher levels of *PD-1*, compared to untreated cells (HC: P=0.007, Naïve: P=0.004 and ART: P=0.002, **Figure 6G**). Similarly, we observed increased levels of *PD-L1* in response to pEV treatment (~4-fold, **Figure 6H**).

We also investigated whether stimulated PD-1+ Jurkat cells differentially expressed *FOXO1*, another miR-139-5p target (42) in response to pEV pre-incubation. Unexpectedly, Jurkat cells treated with pEVs expressed higher levels of *FOXO1* than untreated cells (**Figure 6I**). pEVs from HCs and ART-naïve patients elicited significantly higher levels of *FOXO1* than untreated cells (p=0.03 and 0.04, respectively, **Figure 6I**). Although Jurkat cells pre-incubated with HIV ART pEVs expressed higher levels of *FOXO1* compared to untreated cells, this did not reach significance (**Figure 6I**). Taken together, our results indicate that stimulated Jurkat cells also upregulate miR-139-5p in response to pEV treatment. However, miR-139-5p upregulation did not correspond with reduced expression of *Fos*, *Jun* and *FOXO1* as expected. The increased expression of these genes including *PD-1* and *PD-L1* implies that pEV treatment modulates PD1-PDL1 signalling through miR-139-5p independent mechanisms.

### Inverse Correlation of miR-139-5p With *FOXO1* Expressed by J-Lat 10.6 Cells Treated With pEVs From HIV Patients

**Figure 7:**
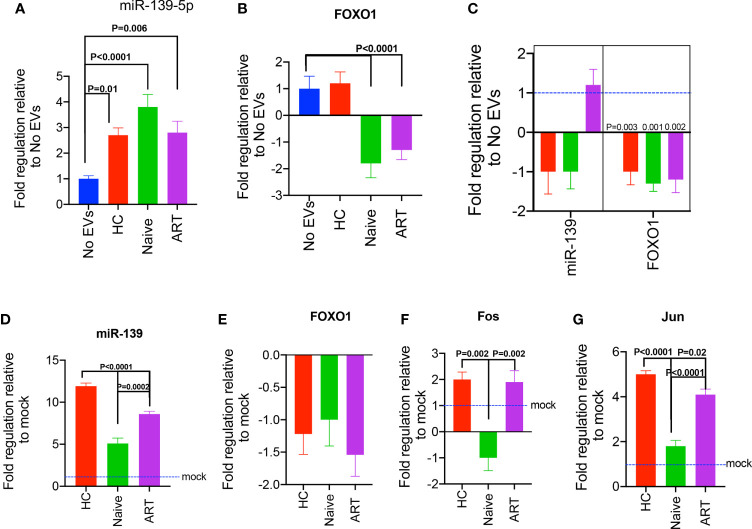
Expression of miR-139-5p and *FOXO1*, by pEV-treated J-Lat 10.6 cells and HIV-1 LAI-infected CD4+ T cells. **(A)** Fold expression of miR-139-5p by unstimulated J-Lat 10.6 cells incubated with the indicated pEVs (5 pEV samples per group) for 72hrs, compared to untreated cells. **(B)** Fold expression of *FOXO1* by J-Lat 10.6 cells treated as stated above. **(C)** Fold expression of miR-139-5p and *FOXO1* by activated CD4+ T cells pre-incubated with the indicated pEVs (5 pEV samples per group) before infection with HIV-1 LAI virus. Data from two independent experiments shown. Error bars indicate mean±SEM. Indicated p-values were obtained from the Dunnett’s multiple comparisons test. The inhibition of miR-139-5p expressed by pEVs from HIV ART-naïve and ART patients, impacts corresponding expression by recipient J-Lat 10.6 cells. **(D)** Fold expression of miR-139-5p by J-Lat 10.6 cells incubated for 72hrs with pEVs hitherto transfected with miR-139-5p inhibitor (500 nM). **(E)** Fold expression of FOXO1, **(F)** Fos and **(G)** Jun by J-Lat 10.6 cells as described above. Error bars indicate mean±SEM. p-values were obtained from the Tukey’s multiple comparisons test.

Since we observed that pEVs from HIV patients expressed miR-139-5p, we decided to evaluate the expression of this miRNA in pEV-treated cells in the context of HIV infection. First, we investigated whether miR-139-5p was differentially expressed by J-Lat 10.6 cells treated with pEVs from HCs, HIV ART-naïve, or patients on ART. We found that J-Lat 10.6 cells treated with pEVs from all groups expressed significantly higher levels of miR-139-5p than untreated cells (**Figure 7A**).

To understand the mechanisms underlying the differential expression of miR-139-5p by pEV-treated J-Lat 10.6 cells, we investigated the expression of one of its target genes, *FOXO1*, which has been verified by computational and experimental methods (42, 44). Furthermore, results from a recent study show that the inhibition of *FOXO1* promotes HIV infection of resting T cells (45).

We observed differential expression of *FOXO1* by J-Lat 10.6 cells treated with pEVs from HIV patients (ART and ART-naïve) in comparison to untreated cells. For instance, there was significant downregulation of *FOXO1* expression by J-Lat 10.6 cells treated with pEVs from HIV ART, and ART-naïve patients, but not HCs (p<0.0001, **Figure 7B**).

We also found that the expression of miR-139-5p by pEV-treated cells inversely correlated with *FOXO1* expression (Pearson’s correlation coefficient for miR-139-5p and *FOXO1*= -1, p=0.06; miR-139-5p, **Supplementary Figure 3E**). These results indicate that pEVs modulate J-Lat 10.6 cells and ensuing viral activation by the interaction between miR-139-5p and its target gene, *FOXO1*.

We also investigated whether pre-incubating CD4^+^ T cells with pEVs before infection with HIV LAI virus can impact the expression of miR-139-5p. We found that CD4^+^ T cells pre-incubated with pEVs from HCs and HIV ART-naïve, but not ART patients, expressed lower levels of miR-139-5p compared to untreated cells, although this was not significant (**Figure 7C**). However, *FOXO1* was significantly downregulated in CD4^+^ T cells that were pre-incubated with pEVs from all groups compared to untreated cells (p=0.003, 0.001, 0.002 for HC, ART-naïve, and ART pEVs respectively, **Figure 7C**). Taken together, these results show that pEV-associated cell and viral activation, particularly by HIV ART and ART-naïve pEVs, may occur through the miR-139-5p/FOXO1 axis.

### Inhibition of pEV-Intrinsic miR-139-5p Results in Differential Expression of *Fos* and *Jun* But not *FOXO1* by Pre-Treated J-Lat 10.6 Cells

To confirm whether our findings were due to pEV-intrinsic miR-139-5p expression, we treated J-Lat 10.6 cells with pEVs hitherto transfected with miR-139-5p inhibitor. We found that J-Lat 10.6 cells pre-incubated with inhibitor-transfected pEVs expressed higher levels of miR-139-5p compared to cells treated with mock-transfected pEVs (**Figure 7D**). It is unclear why *de novo* miR-139-5p expression occurred in J-Lat cells treated with inhibitor-transfected pEVs, and this was significantly higher in cells treated with HC pEVs than HIV ART and ART-naïve pEVs (p<0.0001, p=0.0002 respectively, **Figure 7D** **)**. This observation indicates that ART and ART-naïve pEVs express higher levels of miR-139-5p, which can contribute to endogenous miR-139-5p responses by pre-treated J-Lat 10.6 cells. Unexpectedly, treatment with inhibitor-transfected pEVs did not increase *FOXO1* expression by J-Lat 10.6 cells (**Figure 7E**). On the other hand, J-Lat 10.6 cells treated with inhibitor-transfected pEVs from HIV-ART patients and HCs expressed significantly higher Fos levels than cells treated with HIV naïve pEVs (**Figure 7F**). Furthermore, J-Lat cells expressed significantly higher Jun levels in response to treatment with inhibitor-transfected pEVs (**Figure 7G**). However, inhibitor-transfected HIV naïve pEVs elicited reduced Jun expression compared to HIV ART and HC pEVs (**Figure 7G**). Collectively, these results imply that pEVs, from HC and HIV patients, can contribute to miR-139-5p-associated responses by J-Lat 10.6 cells.

## Discussion

Our observations indicate that pEVs are immunostimulatory particles, which can enhance HIV infection of CD4^+^ T cells and the activation of latent HIV-infected cells. pEVs from both HCs and HIV patients could promote increased HIV infection of activated CD4^+^ T cells, represented by increased cell activation and higher frequencies of p24 expression. Furthermore, incubation of unstimulated, latently infected J-Lat10.6 cells with pEVs resulted in viral activation and hence, GFP expression. Therefore, these results suggest that the interaction of pEVs originating from infected and uninfected cells can promote the infection and dissemination of HIV to uninfected, neighbouring cells. We also found that pEVs from HIV long term non-progressors were also stimulatory, however due to the low number of donors, these results were not included in this study. A number of studies have shown that exosomes secreted by HIV-infected cells contribute to HIV activation, replication and immune pathogenesis, due to their cargoes, which consist of HIV viral accessory proteins as well as inflammatory effectors (9, 46–48). On the other hand, exosomes from uninfected cells can activate latent HIV-1 by facilitating RNA polymerase II loading onto the HIV-promoter in infected cells (49). However, this proof-of-concept study provides a novel insight into how EVs can utilise different mechanisms to promote T cell activation and latent HIV reactivation.

Exosomes and other EVs, play various roles in immune regulation due to the heterogeneity of their contents, which comprise of a myriad of proteins, nucleic acids and lipids (6). Accordingly, EVs can promote both stimulatory and suppressive T cell responses, which often reflects the properties of their parent cells (36, 50–52). Here, we show that pEVs from *healthy settings* and a HIV+ environment, by inducing inflammatory responses, enhance HIV infection of recipient CD4^+^ T cells. Unexpectedly, we found that that the levels of HIV p24 expressed by activated CD4^+^ T cells pre-incubated with pEVs from HIV patients were similar to those pre-incubated with pEVs from healthy subjects. It is, however, unclear whether circulating EVs can stimulate T cells in the absence of infection *in vivo.* Indeed, a number of reports have indicated a role for circulating exosomes in tonic inhibition of peripheral inflammation (53). Furthermore, as pEVs harbour MHC molecules, we cannot rule out the possibility of allogenicity (between pEVs and recipient T cells) contributing to the enhanced activation observed in pEV-treated cells.

We found that prior incubation of ART-naive pEVs with virus induced increased HIV infection than pEVs from HCs, indicating that viral proteins enhance the transmission of HIV by EVs.

Recent strategies to reverse HIV latency include ‘shock and kill’ interventions such as the activation of the NF-κB pathway, a combination of CD8^+^ T cell depletion using antibodies and the activation of IL-15 signalling (2, 3). However, strategies which involve targeting EVs and subsequently their cargoes, have not been explored extensively. Studies have shown that EVs from infected and uninfected HCs can reactivate latent HIV (46, 48, 49). On the contrary, another study has shown that the depletion of EVs favours viral reactivation (54). Hence, more understanding of the mechanisms by which EVs can modulate HIV reactivation will facilitate the development of novel therapeutic approaches.

In this study, we show that the reactivation of latent HIV-infected J-Lat 10.6 cells by pEVs is paralleled by increased miR-139-5p expression and reduced expression of FOXO1. This was unexpected, as observations from tumour studies indicate that miR-139-5p acts as a tumour suppressor (43, 55–57). Nevertheless, the inhibition of FOXO1 in the absence of stimulation has been shown to induce metabolic activation of T cells and their transition from the G0 to the G1 phase (45). Furthermore, FOXO1 inhibition can induce the reactivation of HIV-1 latent proviruses in T cells (45). A direct correlation between the downregulation of FOXO1 expression and increased GFP expression was only observed in response to treatment with pEVs from HIV patients, which suggests that pEV-induced factors, including miR-139-5p, are responsible for reduced FOXO1 expression by J-Lat 10.6 cells in our study.

Notably, we found that increased miR-139-5p expression in response to pEV treatment was peculiar to J-Lat 10.6 cells as this was not observed in activated CD4^+^ T cells pre-incubated with pEVs and infected with HIV. Indeed, T cell activation, through the activity of miR-182 inhibits the expression of FOXO1 to promote clonal expansion (58). Low levels of FOXO1 in activated T cells may indicate a reduced requirement for endogenous miR-139-5p activity. As a consequence, pEV-intrinsic miR-139 may not impact FOXO1 expression in this system. This implies that increased miR-139-5p expression may contribute to FOXO1 downregulation and activation of latently infected J-Lat 10.6 cells. Whether pEVs can utilise this mechanism to facilitate the activation of latent HIV-infected cells such as CD4^+^ T cells and monocytes *in vivo* warrants further investigation.

As EVs harbour various proteins, nucleic acids and lipids, we expected pEVs to modulate cell activation and function through multiple mechanisms. Similar to tumour-derived exosomes, we found that pEVs express PD-1 and PD-L1. Furthermore, we observed that pEVs induced increased miR-139-5p expression in stimulated PD1+ Jurkat cells. However, this was not associated with FOXO1 regulation as pEV-treated Jurkat cells also expressed increased levels of FOXO1. It is likely FOXO1, in conjunction with Fos and Jun, contributed to the increased expression of PD1 and PDL1 by pEV-treated Jurkat cells. This is reminiscent of the observations from a study, in which FOXO1 was found to promote sustained expression of PD1 in CTLs undergoing persistent antigenic stimulation (59). The significance of pEV-induced miR-139-5p expression in this system is however unclear and might be associated with increased expression of FOXO1, Fos, Jun and other target genes beyond the scope of this study.

We also established that miR-139-5p expressed by pEVs from HIV ART-naïve and ART patients, but not HCs, can contribute to endogenous J-Lat 10.6 miR-139-5p activity.

There are two possible reasons for the unexpected reduction in FOXO1 expression by J-Lat cells treated with inhibitor-transfected pEVs. Firstly, increased and high levels of miR-139-5p expressed by J-Lat 10.6 cells irrespective of treatment with inhibitor-transfected pEVs probably counteracted FOXO1 upregulation. Also, other miRNAs expressed by pEVs which target FOXO1 such as miR-132-3p (**Figure 6C**) may have contributed to this response. The latter may also reflect the requirement for significant regulation of FOXO1 expression due to its role in immune homeostasis (60, 61). In contrast, targeting pEV miR-139-5p was sufficient to elicit increased Fos and Jun expression by treated J-Lat 10.6 cells. These observations infer that by transfer of miR-139-5p, which can synergise with cell-intrinsic miR-139-5p expression, pEVs can regulate PD-1 and PD-L1 expression by targeting Fos and Jun. This may have implications for latent HIV-infected memory CD4^+^ T cells, which express high amounts of PD-1 (62).

Collectively, we have shown that pEVs by transferring miR-139-5p, can facilitate the reactivation of latent HIV-infected recipient J-Lat 10.6 cells. Observations from inhibition studies and other *in vitro* assays indicate that the stringent activity of a network of miR-139-5p and other miRNAs are required for the regulation of FOXO1 expression and T cell activation. Furthermore, pEV miR-139-5p by targeting Fos and Jun expression can regulate PD1 and PD-L1 transcription, thus promoting increased T cell activation. It will be imperative to establish whether pEVs or EVs from other sources such as CD4^+^ T cells, monocytes and dendritic cells are a source of this miRNA and can promote the activation of latently infected cells *in vivo*. This will pave the way for the use of extracellular vesicles and other ‘particles’ as therapies to complement current ‘shock and kill’ strategies for the elimination of HIV latency (59). Hence, over-expression and targeting of miR-139-5p-expressing pEVs to HIV reservoirs may contribute to the reversal of HIV latency.

## Data Availability Statement

The original contributions presented in the study are included in the article/**Supplementary Material**. Further inquiries can be directed to the corresponding author.

## Ethics Statement

The studies involving human participants were reviewed and approved by the ethics board at the University of Alberta. The patients/participants provided their written informed consent to participate in this study.

## Author Contributions

IO designed and performed most of the research, analyzed the data and wrote the manuscript. LX performed the Image stream analysis. OO performed western blots, SS assisted in blood sample collection. DP assisted in NTA analysis and provided advice, PG and XS helped with the EM studies. SE conceived the research, secured resources, supervised all of the study and edited the manuscript. All authors contributed to the article and approved the submitted version.

## Funding

This work was supported by a Foundation Scheme grant from the Canadian Institutes of Health Research (CIHR), a CIHR New Investigator Salary Award (all to S.E.).

## Conflict of Interest

The authors declare that the research was conducted in the absence of any commercial or financial relationships that could be construed as a potential conflict of interest.
