# Supplementary material for: Plasma Extracellular Vesicles Enhance HIV-1 Infection of Activated CD4+ T Cells and Promote the Activation of Latently Infected J-Lat10.6 Cells via miR-139-5p Transfer
Source: Front Immunol. 2021 Jun 24;12:697604. doi: 10.3389/fimmu.2021.697604 (PMC8264662; doi:10.3389/fimmu.2021.697604)
Supplement: Supplementary file 1 [file DataSheet_1.pdf]

Supplementary Table 1. Information related to the study subjects

| Patient ID | sex | ART | CD4 count/ml | Viral load (copies/ml) |
|---|---|---|---|---|
| #1 | M | Naïve | 430 | 265037 |
| #3 | M | Naïve | 740 | 26968 |
| #4 | M | Treated | 570 | ND |
| #6 | M | Naïve | 660 | 115740 |
| #12 | M | Treated | 470 | ND |
| #13 | F | Naïve | <10 | 19957 |
| #16 | F | Naïve | 150 | 130570 |
| #28 | M | Naïve | 40 | 41951 |
| #43 | M | Naïve | 270 | 18111 |
| #45 | M | Naïve | 700 | 746 |
| #47 | F | Treated | 190 | ND |
| #50 | M | Treated | 390 | ND |
| #56 | M | Naïve | 710 | 730 |
| #55 | M | Treated | 520 | ND |
| #62 | F | Naïve | 810 | 2186 |
| #63 | M | Treated | 260 | <40 |
| #67 | M | Treated | 830 | ND |
| #70 | M | Treated | 400 | ND |
| #72 | M | Treated | 260 | ND |
| #74 | M | Treated | 700 | <40 |
| #73 | M | Treated | 570 | ND |
| #72 | M | Treated | 260 | ND |
| #71 | M | Treated | 420 | ND |
| #76 | M | Treated | 330 | ND |
| #81 | M | Naïve | 370 | 53770 |
| #82 | F | Naïve | 340 | 145114 |
| #84 | F | Treated | 690 | <40 |
| #85 | M | Treated | 260 | ND |
| #86 | M | Treated | 270 | ND |
| #87 | M | Treated | 110 | ND |
| #88 | F | Treated | 620 | <40 |
| #90 | F | Treated | 770 | ND |
| #98 | F | Treated | 910 | ND |
| #104 | M | Naïve | 400 | 1446308 |
| #128 | M | Treated | 480 | <40 |
| #138 | F | Naïve | 320 | 3282 |
| #140 | M | Naïve | 280 | 492274 |
| #146 | M | Naïve | 260 | 22569 |
| #145 | M | Treated | 480 | ND |

Supplementary Table 1. Information related to the study subjects

Naïve (HIV patients are not on antiretroviral therapy (ART))
Treated (HIV patients on ART)
Male (M), Female (F)
ND (below the detection limit or not detected).
