# Supplementary material for: Plasma Extracellular Vesicles Enhance HIV-1 Infection of Activated CD4+ T Cells and Promote the Activation of Latently Infected J-Lat10.6 Cells via miR-139-5p Transfer
Source: Front Immunol. 2021 Jun 24;12:697604. doi: 10.3389/fimmu.2021.697604 (PMC8264662; doi:10.3389/fimmu.2021.697604)
Supplement: Supplementary file 2 [file DataSheet_2.pdf]

Supplementary Figure 1

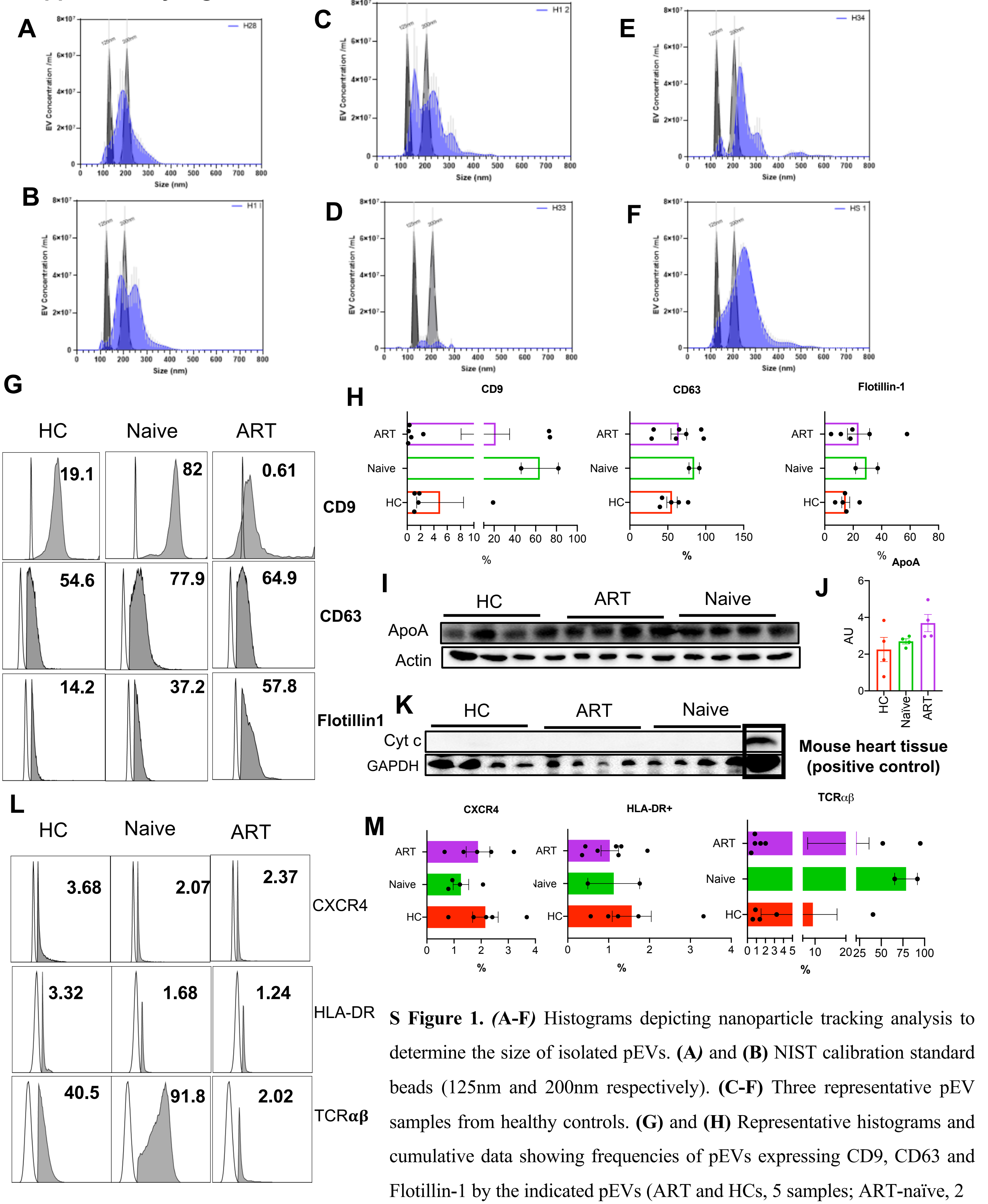

S Figure 1. ***(A-F)*** Histograms depicting nanoparticle tracking analysis to determine the size of isolated pEVs. **(A*)*** and **(B)** NIST calibration standard beads (125nm and 200nm respectively). **(C-F)** Three representative pEV samples from healthy controls. **(G)** and **(H)** Representative histograms and cumulative data showing frequencies of pEVs expressing CD9, CD63 and Flotillin-1 by the indicated pEVs (ART and HCs, 5 samples; ART-naïve, 2 samples). Clear histograms: unstained control. **(I)** and **(J)**, Representative blots and cumulative data showing expression of Apolipoprotein A1 by the indicated pEVs (4 samples per group). Actin – loading control. Data from two experiments shown. **(K)** Representative blots showing expression of cytochrome c by indicated pEVs and positive control, GAPDH-loading control. **(L)** and **(M)**, Representative histograms and cumulative data showing frequencies of pEVs expressing CXCR4, HLA-DR and TCR*α*β by the indicated pEVs. (4 samples per group). Error bars indicate mean±SEM.

Supplementary Figure 2

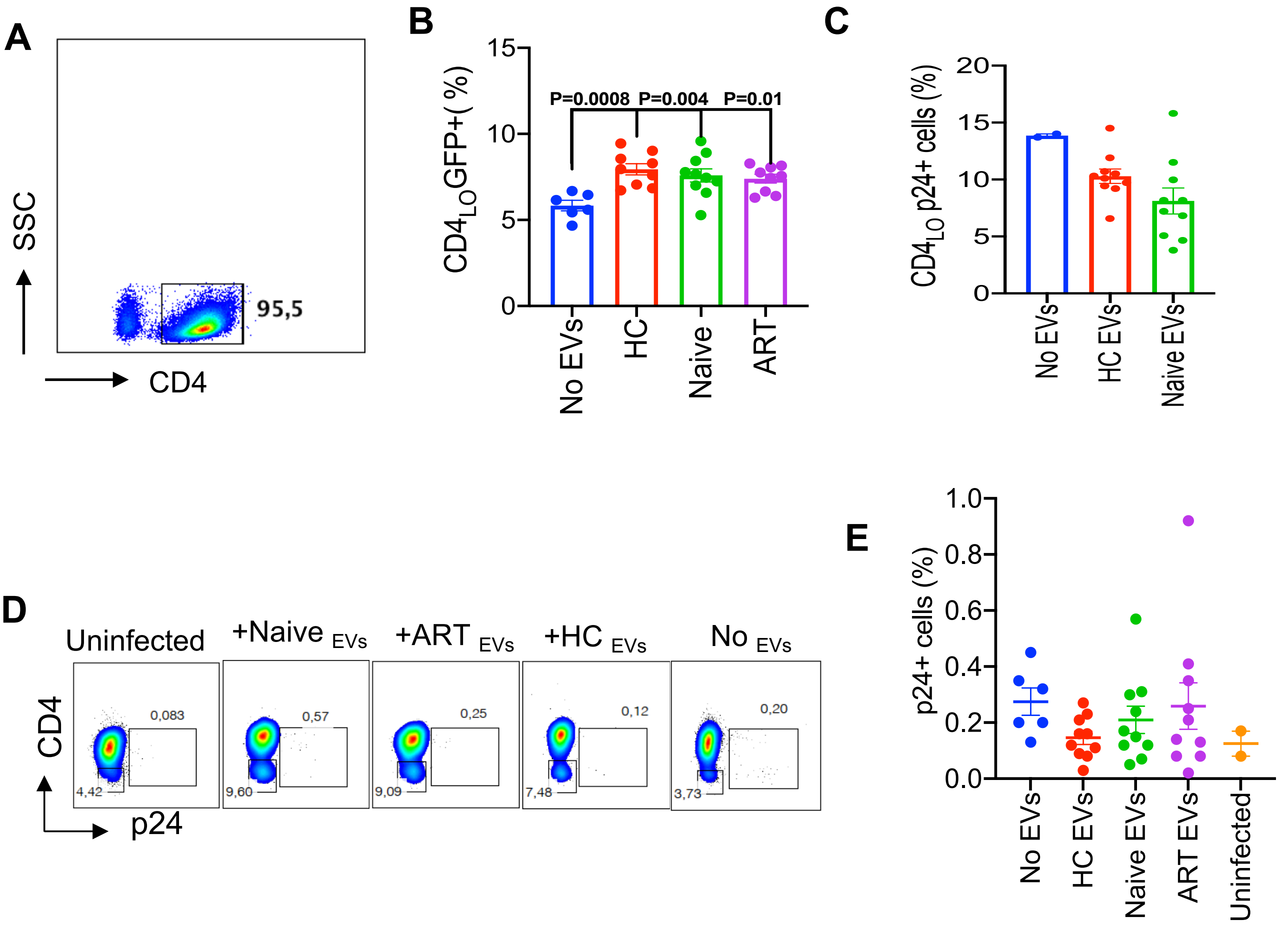

S Figure 2. (A), Representative flow plot showing the purity of isolated CD4+ T cells. **(B)** Bar plots showing cumulative frequencies of CD4 GFP+ cells expressed by J-Lat 10.6 cells pre-incubated with the indicated pEVs and stimulated with anti-CD3 /CD28 for 72hrs. **(C)**, Cumulative data of the frequencies of p24 expressed by activated CD4+ T cells infected with HIV-1 LAI virus followed by incubation with the indicated pEVs. **(D)** Representative plots of p24 expression by unstimulated CD4+ T cells pre-treated with the indicated pEVs and infected with HIV LAI virus. **(E)** Cumulative frequencies of p24+ expressed by unstimulated CD4+ T cells as described in (**D**). Data from 3 individual experiments shown. Data points represent individual samples within pEV groups. Error bars indicate mean±SEM. p-values were obtained from the Dunnett's multiple comparisons test.

Supplementary Figure 3

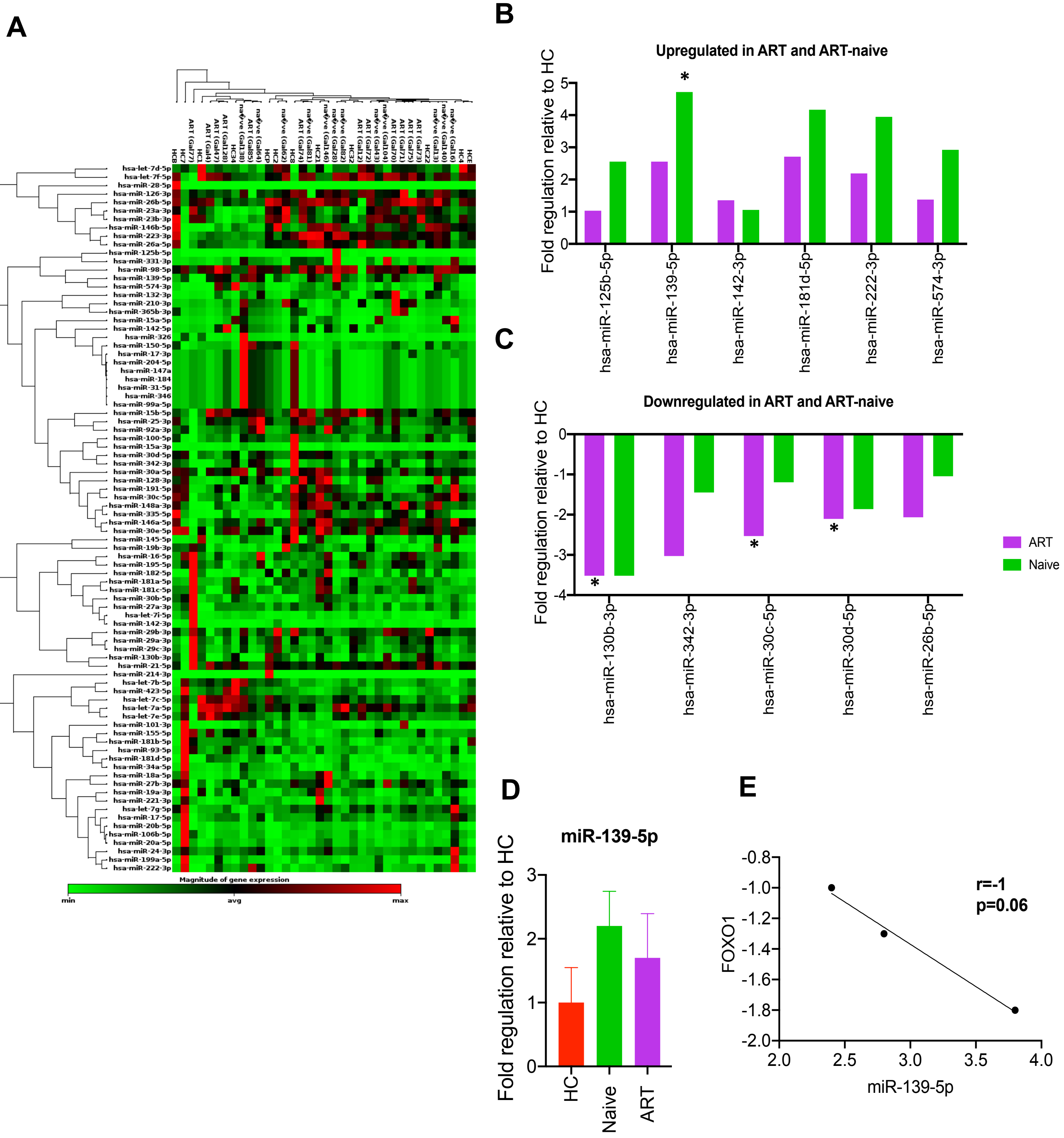

S Figure 3. (A) Hierarchical clustering of miRNAs expressed by pEVs from HCs (H), HIV patients on ART (A), and HIV ART-naïve (N) patients. The heatmap lane for each group represents one pEV sample. (**B**) Fold expression of the indicated upregulated miRNAs by pEVs from HIV patients on ART and ART-naïve patients compared to HCs and **(C),** downregulated miRNAs. ** p-value= 0.003, *p-value=0.02. **(D)** Fold expression of miR-139-5p by HIV ART-naïve, and ART pEVs, relative to expression by pEVs from HCs (12 samples per group). Error bars indicate mean±SEM. Pearson correlation between the mean expression of FOXO1 **(E)** with miR-139-5p expressed by pEV-treated J-Lat 10.6 cells (n=4-5, per pEV group).
